# Perinatal Pet Exposure, Faecal Microbiota, and Wheezy Bronchitis: Is There a Connection?

**DOI:** 10.1155/2013/827934

**Published:** 2013-01-09

**Authors:** Merja Nermes, Katri Niinivirta, Lotta Nylund, Kirsi Laitinen, Jaakko Matomäki, Seppo Salminen, Erika Isolauri

**Affiliations:** ^1^Department of Pediatrics, Turku University Hospital, Kiinamyllynkatu 4-8, 20520 Turku, Finland; ^2^Department of Clinical Sciences, University of Turku, Turku, Finland; ^3^Functional Foods Forum, University of Turku, Turku, Finland; ^4^Institute of Biomedicine, University of Turku, Turku, Finland

## Abstract

*Background*. The hygiene hypothesis suggests that high hygiene standards have led to an immune dysfunction and an increase in allergic diseases. Farming-related exposures are associated with a decreased risk of asthma. Since the gut microbiota may be a pivotal component in the hygiene hypothesis, we studied whether perinatal exposure to pets, doctor's diagnosed wheezy bronchitis (WB), and compositional changes in the gut microbiota are interrelated among urban infants. *Methods*. Data were collected prospectively from a mother-infant nutrition study. Data on perinatal pet ownership, WB, and the microbiota composition of faecal samples of the infants assessed by quantitative PCR at 1 month were compared. *Results*. None of the 30 infants exposed to pets had suffered from WB by 24 months, whereas 15 of the 99 (15%) nonexposed infants had had WB (*P* = 0.03). The counts of *Bifidobacterium longum* were higher in samples (*n* = 17) from nonwheezing infants with pet exposure compared to those (*n* = 10) in wheezing infants without pet exposure (8.59/10.44 versus 5.94/9.86, resp. (median/upper limit of range, bacteria(log)/g of stool); *P* = 0.02). *B. breve* was more abundant in the wheezing infants (*P* = 0.02).

## 1. Introduction

The development of asthma and allergic diseases is a result of complex interactions between genetic predisposition and multiple environmental influences [1]. The hygiene hypothesis, first proposed by Strachan in 1989 [2] and subsequently supported by epidemiological studies [3], suggests that the higher hygiene standards adopted during the last few decades have led to dysfunction in the immune system, giving rise to allergic and autoimmune diseases as seen especially in affluent societies. Modern infants living in the developed countries may thus lack stimulation of the mucosal immune system sufficient to generate a tolerogenic immune milieu and be prone to develop diseases of inflammatory origin. In support of such a conception, differences in the neonatal gut microbiota have been shown to precede the development of atopy; for example, infants in whom atopy is developing harbour fewer bifidobacteria than their nonatopic peers [4]. The original hygiene hypothesis has been extended to a microbiota hypothesis emphasising the importance of the indigenous intestinal microbiota [5]. 

Farming-related exposures such as exposure to animal sheds and raw milk consumption have been shown to protect against the development of asthma and allergies [3]. Children living on farms are exposed to a wide range of microbes, and the diversity of microbial exposure is inversely associated with the risk of asthma [6]. Microbial communities in the household have also been shown to be significantly impacted by the presence of indoor pets [7, 8]. In epidemiological studies exposure to indoor cats [9] and dogs [10] early in childhood has been linked with a diminished incidence of asthma and decreased respiratory infectious disease morbidity [11]. Evidence has been provided for the role of the innate and adaptive immune systems in mediating these protective effects [12, 13]. 

We hypothesized that the composition of the microbiome in the homes of families with indoor pets may impact the composition of the infant gut microbiota and shape the immune responses, providing protection against wheezing illness in infancy. Therefore we investigated the interrelationship between perinatal pet exposure, infant wheezy bronchitis as diagnosed by a physician, and the composition of the faecal microbiota. 

## 2. Subjects and Methods

### 2.1. Subjects and Study Design

This study was part of a randomized mother-infant nutrition and probiotic study (section 3 in, http://www.clinicaltrials.gov/ct/gui/show/NCT00167700), described in detail elsewhere [14]). In brief, altogether 256 pregnant women from families in which at least one member had asthma or an allergic disease were recruited in the first trimester of pregnancy, at the first visit to maternal welfare clinics in Turku and neighbouring areas in South-West Finland. At entry, the women were randomized into three groups: (1) a group receiving probiotics and dietary counselling (*n* = 85) provided by a nutritionist and designed to modify dietary intake according to the recommendations at the time of study initiation [15, 16], (2) a group receiving placebo and dietary counselling (*n* = 86), and (3) a group receiving placebo without dietary counselling (*n* = 85). In groups 1 and 2, the mothers were randomized to receive probiotics or placebo in double-blind manner. In group 3, the mothers received placebo single-blinded. To strengthen the dietary course and to demonstrate sources of favourable fat and fibre content, various food products available on the market were provided for use at home. 

The probiotics used in the study were *Lactobacillus rhamnosus* GG (ATCC 53103, Valio Ltd, Helsinki, Finland) and *Bifidobacterium* lactis (Chr. Hansen, Horsholm, Denmark) at a dose of 10^10^ cfu/day each. Maternal probiotic consumption continued until the cessation of exclusive breastfeeding or until the infant was 6 months of age. Written informed consent was obtained from the participants, and the Ethics Committee of the Hospital District of South-West Finland approved the study.

Altogether 129 mothers with data on indoor pet keeping, mainly cats and dogs, were included in this part of the study. Characteristics of the study infants are set out in Table 1. Clinical examinations of the infants were carried out and data collected at the ages of 1, 6, 12, and 24 months. An infant was confirmed as having had doctor's diagnosed wheezy bronchitis if at least one period of wheezing, cough, and breathing difficulty could be verified in the Hospital District medical records by the age of 24 months. Data on demographic aspects and the medical history of mother and child were collected using semi-structured interviews throughout the study. 

#### 2.1.1. PCR Analysis of Faecal Samples

*DNA Extraction from Faecal Samples.* Faecal samples at 1 month of age were obtained from 17 nonwheezing, pet-exposed and 10 wheezing, nonexposed infants. The samples (0.3–0.6 g) were diluted 1/10 with phosphate buffered saline and homogenized 30 s with glass beads (5 mm and 1.5 mm in diameter). This homogenate was further treated with glass beads (0.1 mm in diameter) for 1 min, and 200 *μ*L of faecal homogenate was used for DNA extraction with QIAmp DNA Stool Mini Kit (Qiagen, Hilden, Germany). Extracted DNA was eluted in 200 *μ*L of AE buffer (Qiagen) and stored at −20°C until analyses.

*Quantitative PCR.* Quantitative PCR (qPCR) analysis of *Bifidobacterium* genus and the most important species present in the human gut was carried out in an Applied Biosystems 7300 Fast Real-Time PCR System in a 96-well format and using SYBR Green chemistry (SYBR Green PCR Master Mix, Applied Biosystems, USA). The primers, their specificities, and the PCR thermocycling conditions are presented in Table 2. Standards for qPCR were prepared as described in Nermes et al. [17]. Samples were analysed in duplicate in at least two independent runs. 

### 2.2. Skin Prick Tests

To study the presence of atopy in the study infants, that is, IgE-mediated sensitization to environmental allergens, skin prick tests (SPTs) were performed at 24 months. The antigens tested included fresh cow's milk (3.3 g/100 g), raw hen's egg white, wheat and rice flour both diluted 1/10 (w/v) with 0.9% (w/v) sodium chloride, and gliadin 1 mg/mL (Hospital Pharmacy of Turku University Hospital, Turku, Finland). Further, cod, soya bean, birch, six grasses, cat, dog, *Dermatophagoides pteronyssinus* allergen Der p1 (ALK, Horsholm, Denmark), and latex (Laboratorium Stallergenes SA, Anthony cedex, France) were tested. Histamine dihydrochloride (10 mg/mL, ALK, Horsholm, Denmark) was used as positive control and physiological saline as negative. The perpendicular diameters of each SPT reaction were recorded after 15 min, and the results expressed as mean weal diameter. Diameters ≥3 mm were considered positive. An infant was considered atopic if at least one of the tested allergens was positive.

#### 2.2.1. Statistical Methods

The association of pet exposure with WB was calculated using exact logistic regression analysis. Other associations between predictors and dichotomous response variables were studied with univariate logistic regression analysis. Mann-Whitney *U* test was used for comparison of continuous variables such as results of PCR analysis of faecal samples between two groups. Statistical analyses were performed using SAS for Windows version 9.2. *P*  values < 0.05 were considered statistically significant.

## 3. Results

None of the 30 infants who were perinatally exposed to pets had suffered from WB by 24 months, whereas 15 (15.3%) of the nonexposed infants had (*P* = 0.03). Maternal asthma, maternal atopy, parental smoking, duration of breast feeding, study group, number of siblings, and gender were considered as potential confounders of the association between pet exposure and WB; however, no significant associations were found between pet exposure and potential confounders. Eight (26.7%) of the pet-exposed and 42 (42.4%) of the nonexposed infants were atopic by 24 months (*P* = 0.12). 

Faecal samples at the age of 1 month were obtained from 17 nonwheezing, pet-exposed infants and from 10 wheezing, nonexposed infants. *B. longum* was more abundant in samples from the nonwheezing pet-exposed than in those from the wheezing nonexposed infants (*P* = 0.02), whereas *B. breve *was detected in higher amounts in the latter group (Table 3). The counts of *B. breve* were above the detection limit in 1/17 (6.9%) of the samples of the exposed and in 5/10 (50.0%) of the nonexposed infants.

## 4. Discussion

To our knowledge, this study is the first to show that perinatal exposure to indoor pets among urban infants, protection from wheezy bronchitis, and compositional differences in the gut microbiota are associated. Although farming-related exposures and keeping indoor pets have already been shown to confer protection against asthma and wheeze, the mechanisms of this protective effect have remained unravelled, even though they have been suggested to be mediated via innate immunity [27]. CD 14 and toll-like receptors (TLRs), which recognize pathogen-associated molecular patterns at the interfaces of an organism with the environment, play a pivotal role in triggering the responses of innate immunity. Environmental exposures which are rich in microbes and where such microbes are encountered during pregnancy or early life have been reported to be associated with upregulation of mRNA expression of innate immune receptors [28, 29] and a decreased risk of asthma and allergic disease [6, 30]. Our results demonstrate significant differences in the gut microbiota, as indicated by differences in bifidobacteria composition, between the nonwheezing and wheezing infants depending on the presence of pets in the household. These results are in agreement with earlier reports on the microbiota and innate immunity and link results from epidemiological and experimental studies.

Murray et al. [31] evaluated the composition of faecal microbiota in sensitized wheezy and nonsensitized non-wheezy children at 4 years of age and did not find any differences between the two groups. However, the setting of their study was different from that of ours. The children in this study were younger. The composition of faecal microbiota was investigated at the age of 1 month, that is, earlier in life, and it may be possible that the differences disappear later. Further, in the study of Murray et al. the children had a history of parentally reported wheeze, whereas the children of this study had doctor's diagnosed wheeze which implies that the disease may have been more severe. 

Bifidobacteria have been demonstrated to be important for the healthy development of the gut microbiota in infants, and low levels of *Bifidobacteria* have been associated with the subsequent development of allergic disease [4, 32]. In fact, *Bifidobacteria* are the most abundant members and are regarded as key biological markers of the gut microbiota of the healthy breast-fed infants [33]. Our results indicate that specific *Bifidobacterium *species are associated with distinct outcomes; *Bifidobacterium longum* links the potentially protective effect of pet exposure from WB, whereas *Bifidobacterium breve* is interrelated with lack of pet exposure and WB. This finding of different outcomes is in accordance with the previous observation where *Bifidobacterium longum* conferred protection and *Bifidobacterium breve* heightened risk of crying and fussing during the first months of life of an infant [34]. There are also data to show that individual *Bifidobacterium* species exert distinct effects on immunity and disease risk [35]. An experimental study in gnotobiotic mice gave some indication of the different effects of *Bifidobacterium longum* and *Bifidobacterium breve* on the developing immune system [36]. *Bifidobacterium longum* strains isolated from infants' faecal microbiota were able to induce strain-specific effects on systemic and intestinal immunity whereas *Bifidobacterium breve* strains were found to have little effect on the immune system [36].

In conclusion, this study showed in an urban setting that perinatal pet exposure, compositional differences in the gut microbiota, and protection from early wheezing are interrelated. Further research is needed to elucidate the composition of microbial environments associated with indoor pet keeping, their impact on the developing gut microbiota of an infant, and the mode of action in inducing disease-modifying effects.

## Figures and Tables

**Table 1:** Characteristics of the study patients.

Gender	
male, *n* (%)	61 (47.3)
female, *n* (%)	68 (52.7)
Birth weight, g, mean; range	3574; 2090–4660
Mode of delivery*	
vaginal birth, *n* (%)	103 (85.1)
caesarean section, *n* (%)	18 (14.9)
Maternal smoking during pregnancy	
yes, *n* (%)	2 (1.6)
no, *n* (%)	127 (98.4)
Older siblings	
yes, *n* (%)	50 (39.8)
no, *n* (%)	79 (61.2)
Duration of breastfeeding, mean; range (mo)	9.6; (0.5–36)

*Data of 8 mothers missing.

**Table 2:** Primers used in quantitative PCR.

Target	Primers	Sequence from 5′ to 3^′a^	Annealing temperature	Reference
*Akkermansia muciniphila *	AM1	CAGCACGTGAAGGTGGGGAC	50°C	[18]
	AM2	CCTTGCGGTTGGCTTCAGAT		
*Bifidobacterium* genus	Bifido5′	GAT TCT GGC TCA GGA TGA ACG C	60°C	[19, 20]
	Bifido3′	CTG ATA GGA CGC GAC CCC AT		
*Bifidobacterium adolescentis *	Ado-Ang5′	GGA TCG GCT GGA GCT TGC TCC G	63°C	[21]
	Ado3′	CCC CGA AGG CTT GCT CCC AGT		
*Bifidobacterium bifidum *	Bifidum5′	TGA CCG ACC TGC CCC ATG CT	61°C	[21]
	Bifidum3′	CCC ATC CCA CGC CGA TAG AAT		
*Bifidobacterium breve *	Breve5′	AAT GCC GGA TGC TCC ATC ACA C	62°C	[21]
	Breve3′	GCC TTG CTC CCT AAC AAA AGA GG		
*Bifidobacterium catenulatum *group	Caten5′	GCC GGA TGC TCC GAC TCC T	64°C	[21]
	Caten3′	ACC CGA AGG CTT GCT CCC GAT		
*Bifidobacterium longum *group	Longum5′	TTC CAG TTG ATC GCA TGG TCT TCT	65°C	[21]
	Longum3′	GGC TAC CCG TCG AAG CCA CG		
*Clostridium coccoides *group	g-Ccoc-F	AAA TGA CGG TAC CTG ACT AA	53°C	[22]
	g-Ccoc-R	CTT TGA GTT TCA TTC TTG CGA A		
*Clostridium difficile *	Cdif-F	TTG AGC GAT TTA CTT CGG TAA AGA	62°C	[23]
	Cdif-R	TGT ACT GGC TCA CCT TTG ATA TTY A		
*Clostridium leptum *subgroup	sg-Clept-F	GCA CAA GCA GTG GAG T	60°C	[24]
	sg-Clept-R3	CTT CCT CCG TTT TGT ACC		
*Clostridium perfringens *	CPerf165F	CGC ATA AY^b^G TTG AAA GAT GG	64°C	[25]
	CPerf269R	CCT TGG TAG GCC GTT ACC C		
*Staphylococcus aureus nuc *gene	NUC1	GCG ATT GAT GGT GAT ACG GTT	55°C	[26]
	NUC2	AGC CAA GCC TTG ACG AAC TAA AGC		

^a^R represents a (A/G) wobble nucleotide; Y represents a (C/T) wobble nucleotide.

^b^C in the reference was replaced by Y to detect better different *C. perfringens* strains.

**Table 3:** Results of qPCR analysis of the faecal samples (bacteria(log)/g of faeces) from pet-exposed, non-wheezing (nWB) infants (*n* = 17) and non-exposed, wheezing (WB) infants (*n* = 10) at one month of age.

	Median	Range	Samples (*n*); ND^†^	*P* value
*Bifidobacterium *sp.				
nWB	9.57	6.56–10.29	0	0.06
WB	7.41	6.38–10.65	0	
*B. longum *				
nWB	8.59	ND–10.44	2	0.02*
WB	5.94	ND–9.86	4	
*B. breve *				
nWB	ND	ND–9.96	16	0.02*
WB	ND	ND–9.38	5	
*B. bifidum *				
nWB	ND	ND–9.85	12	0.30
WB	ND	ND–6.15	5	
*B. adolescentis *				
nWB	ND	ND–8.30	13	0.10
WB	ND	ND	10	
*B. catenulatum *				
nWB	ND	ND–9.54	9	0.70
WB	ND	ND–10.10	6	
*Staph. aureus *				
nWB	ND	ND–6.65	12	0.80
WB	ND	ND–6.00	7	
*Akkermansia muciniphila *				
nWB	ND	ND–5.36	15	0.25
WB	ND	ND–4.70	7	
*Clostridium coccoides* group				
nWB	ND	ND–7.59	9	0.80
WB	ND	ND–8.97	7	
*Clostridium leptum* subgroup				
nWB	ND	ND–8.78	9	0.78
WB	ND	ND–5.11	6	
*Clostridium perfringens *				
nWB	ND	ND–9.00	14	0.45
WB	ND	ND–5.09	7	
*Clostridium difficile *				
nWB	ND	ND–8.59	14	0.87
WB	ND	ND–7.96	7	

*Significant differences between pet-exposed non-wheezing and non-exposed wheezing infants.

Mann-Whitney *U* test.

^†^Bacteria not detected.

## References

[1] Ho SM (2010). Environmental epigenetics of asthma: an update. *Journal of Allergy and Clinical Immunology*.

[2] Strachan DP (1989). Hay fever, hygiene, and household size. *British Medical Journal*.

[3] von Mutius E, Vercelli D (2010). Farm living: effects on childhood asthma and allergy. *Nature Reviews Immunology*.

[4] Kalliomäki M, Kirjavainen P, Eerola E, Kero P, Salminen S, Isolauri E (2001). Distinct patterns of neonatal gut microflora in infants in whom atopy was and was not developing. *Journal of Allergy and Clinical Immunology*.

[5] Rautava S, Ruuskanen O, Ouwehand A, Salminen S, Isolauri E (2004). The hygiene hypothesis of atopic disease—an extended version. *Journal of Pediatric Gastroenterology and Nutrition*.

[6] Ege MJ, Mayer M, Normand AC (2011). Exposure to environmental micro-organisms and childhood asthma. *The New England Journal of Medicine*.

[7] Maier RM, Palmer MW, Andersen GL (2010). Environmental determinants of and impact on childhood asthma by the bacterial community in household dust. *Applied and Environmental Microbiology*.

[8] Fujimura KE, Johnson CC, Ownby DR (2010). Man’s best friend? the effect of pet ownership on house dust microbial communities. *Journal of Allergy and Clinical Immunology*.

[9] Oberle D, von Mutius E, von Kries R (2003). Childhood asthma and continuous exposure to cats since the first year of life with cats allowed in the child’s bedroom. *Allergy*.

[10] Remes ST, Castro-Rodriguez JA, Holberg CJ, Martinez FD, Wright AL (2001). Dog exposure in infancy decreases the subsequent risk of frequent wheeze but not of atopy. *Journal of Allergy and Clinical Immunology*.

[11] Bergroth E, Remes S, Pekkanen J, Kauppila T, Büchele G, Keski-Nisula L (2012). Respiratory tract illnesses during the first year of life: effect of dog and cat contacts. *Pediatrics*.

[12] Pfefferle PI, Büchele G, Blümer N (2010). Cord blood cytokines are modulated by maternal farming activities and consumption of farm dairy products during pregnancy: the PASTURE study. *Journal of Allergy and Clinical Immunology*.

[13] Roduit C, Wohlgensinger J, Frei R (2011). Prenatal animal contact and gene expression of innate immunity receptors at birth are associated with atopic dermatitis. *Journal of Allergy and Clinical Immunology*.

[14] Laitinen K, Poussa T, Isolauri E (2009). Probiotics and dietary counselling contribute to glucose regulation during and after pregnancy: a randomised controlled trial. *British Journal of Nutrition*.

[15] Nordic Working Group on Diet and Nutrition (1996). Nordic nutritional recommendations. *Scandinavian Journal of Nutrition*.

[16] Becker W, Lyhne N, Pedersen AN (2004). Nordic nutrition recommendations 2004—integrating nutrition and physical activity. *Scandinavian Journal of Nutrition*.

[17] Nermes M, Kantele JM, Atosuo TJ, Salminen S, Isolauri E (2011). Interaction of orally administered Lactobacillus rhamnosus GG with skin and gut microbiota and humoral immunity in infants with atopic dermatitis. *Clinical and Experimental Allergy*.

[18] Collado MC, Derrien M, Isolauri E, de Vos WM, Salminen S (2007). Intestinal integrity and Akkermansia muciniphila, a mucin-degrading member of the intestinal microbiota present in infants, adults, and the elderly. *Applied and Environmental Microbiology*.

[19] Kaufmann P, Pfefferkorn A, Teuber M, Meile L (1997). Identification and quantification of Bifidobacterium species isolated from food with genus-specific 16S rRNA-targeted probes by colony hybridization and PCR. *Applied and Environmental Microbiology*.

[20] Marteau P, Pochart P, Doré J, Béra-Maillet C, Bernalier A, Corthier G (2001). Comparative study of bacterial groups within the human cecal and fecal microbiota. *Applied and Environmental Microbiology*.

[21] Rinne MM, Gueimonde M, Kalliomäki M, Hoppu U, Salminen SJ, Isolauri E (2005). Similar bifidogenic effects of prebiotic-supplemented partially hydrolyzed infant formula and breastfeeding on infant gut microbiota. *FEMS Immunology and Medical Microbiology*.

[22] Matsuki T, Watanabe K, Fujimoto J (2002). Development of 16S rRNA-gene-targeted group-specific primers for the detection and identification of predominant bacteria in human feces. *Applied and Environmental Microbiology*.

[23] Penders J, Vink C, Driessen C, London N, Thijs C, Stobberingh EE (2005). Quantification of Bifidobacterium spp., Escherichia coli and Clostridium difficile in faecal samples of breast-fed and formula-fed infants by real-time PCR. *FEMS Microbiology Letters*.

[24] Matsuki T, Watanabe K, Fujimoto J, Takada T, Tanaka R (2004). Use of 16S rRNA gene-targeted group-specific primers for real-time PCR analysis of predominant bacteria in human feces. *Applied and Environmental Microbiology*.

[25] Wise MG, Siragusa GR (2005). Quantitative detection of Clostridium perfringens in the broiler fowl gastrointestinal tract by real-time PCR. *Applied and Environmental Microbiology*.

[26] Brakstad OG, Aasbakk K, Maeland JA (1992). Detection of Staphylococcus aureus by polymerase chain reaction amplification of the nuc gene. *Journal of Clinical Microbiology*.

[27] Loss G, Bitter S, Wohlgensinger J (2012). Prenatal and early-life exposure alter expression of innate immunity genes: the PASTURE cohort study. *Journal of Allergy and Clinical Immunology*.

[28] Lauener RP, Birchler, Adamski J (2002). Expression of cD14 and Toll-like receptor 2 in farmers’ and non-farmer’s children. *The Lancet*.

[29] Ege MJ, Frei R, Bieli C (2007). Not all farming environments protect against the development of asthma and wheeze in children. *Journal of Allergy and Clinical Immunology*.

[30] Genuneit J (2012). Exposure to farming environments in childhood and asthma and wheeze in rural populations: a systematic review with meta-analysis. *Pediatric Allergy and Immunology*.

[31] Murray CS, Tannock GW, Simon MA (2005). Fecal microbiota in sensitized wheezy and non-sensitized non-wheezy children: a nested case-control study. *Clinical and Experimental Allergy*.

[32] Sjögren YM, Jenmalm MC, Böttcher MF, Sjökrstén B, Sverremark-Ekström E (2009). Altered early infant gut microbiota in children developing allergy up to 5 years of age. *Clinical & Experimental Allergy*.

[33] Favier CF, Vaughan EE, de Vos WM, Akkermans ADL (2002). Molecular monitoring of succession of bacterial communities in human neonates. *Applied and Environmental Microbiology*.

[34] Pärtty A, Kalliomäki M, Endo A, Salminen S, Isolauri E (2012). Compositional development of Bifidobacterium and Lactobacilllus microbiota is linked with crying and fussin in early infancy. *PLoS One*.

[35] Young SL, Simon MA, Baird MA (2004). Bifidobacterial species differentially affect expression of cell surface markers and cytokines of dendritic cells harvested from cord blood. *Clinical and Diagnostic Laboratory Immunology*.

[36] Ménard O, Butel MJ, Gaboriau-Routhiau V, Waligora-Dupriet AJ (2008). Gnotobiotic mouse immune response induced by Bifidobacterium sp. strains isolated from infants. *Applied and Environmental Microbiology*.

